# Evolutionary History and Phylogeny of Biochemical Functions of Hypericin and Hyperforin in Hypericum spp

**DOI:** 10.1192/j.eurpsy.2025.2069

**Published:** 2025-08-26

**Authors:** D. J. Cox, R. Maldonado-Puebla, B. Carr

**Affiliations:** 1 Psychiatry, University of Florida, Gainesville, United States

## Abstract

**Introduction:**

Hypericum spp., particularly Hypericum perforatum (such as St. John’s Wort), produce hypericin and hyperforin, secondary metabolites that play critical roles in the plant’s defense mechanisms. These compounds, characterized by their polycyclic and lipophilic properties, have evolved to deter herbivores and protect against pathogens. Understanding the evolutionary pressures that shaped these compounds enhances our knowledge of their biochemical roles.

**Objectives:**

This review aims to synthesize current knowledge on the evolutionary development of hypericin and hyperforin within the Hypericum genus, focusing on how these metabolites evolved to fulfill defensive ecological functions.

**Methods:**

A comprehensive literature review was conducted, examining phylogenomic studies, structural analyses, and biochemical research related to the biosynthesis of hypericin and hyperforin. We reviewed relevant phylogenetic data to understand the diversification of these compounds across Hypericum spp.

**Results:**

The literature supports that hypericin and hyperforin evolved in response to selective pressures during the Cretaceous-Paleogene boundary, with their complex polycyclic aromatic structures optimized for defense. These structures, which include conjugated π-systems, are central to the compounds’ ability to deter herbivores and resist pathogens, reflecting an evolutionary adaptation that is conserved across the genus.

**Conclusions:**

The evolution of hypericin and hyperforin within Hypericum spp. is a prime example of how secondary metabolites serve dual purposes in nature and human use. The phylogenetic and biochemical insights reviewed highlight the importance of these compounds as both ecological defenses and pharmacologically active agents.

**Disclosure of Interest:**

None Declared

